# Use of the Complex Zeros of the Partition Function to Investigate the Critical Behavior of the Generalized Interacting Self-Avoiding Trail Model

**DOI:** 10.3390/e21020153

**Published:** 2019-02-05

**Authors:** Damien Foster, Ralph Kenna, Claire Pinettes

**Affiliations:** 1 Fluid and Complex Systems Research Centre, Faculty of Engineering, Environment and Computing, Coventry CV1 5FB, UK; 2 Laboratoire de Physique Théorique et Modélisation (CNRS UMR 8089), Université de Cergy-Pontoise, 2 ave A. Chauvin, 95302 Cergy-Pontoise CEDEX, France

**Keywords:** self-avoiding walks, phase transitions, complex zeros, critical exponents, polymers, frustration

## Abstract

The complex zeros of the canonical (fixed walk-length) partition function are calculated for both the self-avoiding trails model and the vertex-interacting self-avoiding walk model, both in bulk and in the presence of an attractive surface. The finite-size behavior of the zeros is used to estimate the location of phase transitions: the collapse transition in the bulk and the adsorption transition in the presence of a surface. The bulk and surface cross-over exponents, ϕ and $\phi_{S}$, are estimated from the scaling behavior of the leading partition function zeros.

## 1. Introduction

**Figure 1:**
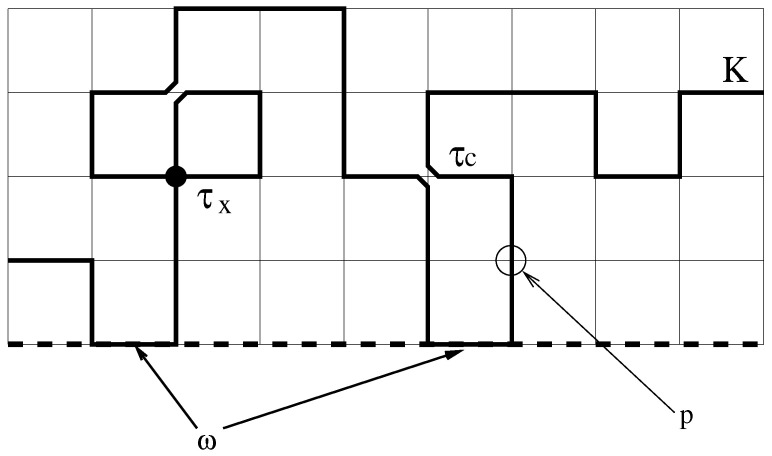
The generalized trail model. Weights $\tau_{C}$ and $\tau_{X}$ are introduced for collisions and crossings at a site, respectively, and weights *p* and ω are introduced to model chain stiffness and attraction with a surface (dotted line).

The interacting self-avoiding walk (ISAW) is the canonical lattice model for a polymer in dilute solution, which takes solvent quality into account [1,2]. In this set-up, the polymer is modelled by a random walk on a lattice. The strong entropic repulsion in the polymer is modelled by adding the constraint that lattice bonds and sites may only be visited once. The quality of the solvent, determined by the difference between the monomer-monomer affinity and the monomer-solvent affinity, is modelled by introducing an attractive interaction energy, ε<0, between pairs of non-consecutive, nearest-neighbor visited sites [3]. This model has been much studied, and is now believed to be well understood, particularly in two dimensions [1]. The arguments that led to the ISAW model are quite general (an excluded volume interaction and short-ranged attraction) and are satisfied by other models, in particular, the interacting self-avoiding trail (ISAT) model [4] and the vertex-interacting self-avoiding walk (VISAW) model (which corresponds to the n→0 limit of an O(n) model proposed by Blöte and Nienhuis) [5]. In both models the self-avoidance is modelled at the level of the lattice bonds (the walk is only allowed to visit a lattice bond once). The walk may visit lattice sites more than once (up to twice on the square lattice), and an interaction energy is attributed to multiply visited sites. The difference between the two models is that the ISAT walk is allowed to cross at a site while the VISAW is not. These two models are sub-models of a generalized ISAT model where the crossings and collisions have different energies $\varepsilon_{c}$ for collisions and $\varepsilon_{x}$ for crossings. In what follows, it is convenient to think in terms of Boltzmann weights $\tau_{x}=\exp\left(-\beta \varepsilon_{x}\right)$ for crossings and $\tau_{C}=\exp\left(-\beta \varepsilon_{c}\right)$ for collisions (β=1/kT with *k* the Boltzmann constant). This model can be generalized further to include a stiffness, realized by associating a Boltzmann weight *p* with sites where the walk carries on in the same direction (p>1 encourages straight sections over corners while p<1 favors corners). For the model see Figure 1. The weights are chosen to be consistent with Blöte and Nienhuis [5]. We also consider the situation where the trail can interact with a surface. When a surface is present, an additional surface weight ω≥1 is added for every contact with the surface. The weight ω drives an adsorption transition at a particular value $\omega_{c}\left(\tau_{x},\tau_{c}\right)$.

It is often convenient to think of these walk models in the grand-canonical ensemble, where a Boltzmann weight linked to the chemical potential required to add a step to the walk, otherwise called the fugacity, *K* is introduced. This fugacity controls the average length of the chain in the grand-canonical ensemble. For a given value of the other weights, the length of the chain increases as *K* is increased from zero until, at some value of $K=K^{⋆}$ the length of the chain diverges. For small values of $\tau_{c/x}$ this happens smoothly in a second order transition, while for larger values of $\tau_{c/x}$ this happens discontinuously (indicating a first-order transition in length). The surface defined by $K^{⋆}\left(\tau_{c},\tau_{x}\right)$ separates a finite-density region ($K>K^{⋆}$) from a zero-density phase ($K<K^{⋆}$). For the ISAW model, the upper region is one finite-density critical phase and there is a special point separating the second order transition line from the first-order transition line. This point is a tricritical point, called the Θ-point, and occurs at a particular value of $\tau_{\Theta }=1.93\pm 0.03$ [6]. If we briefly consider what happens for very long (infinite) walks of fixed length, then we can see that as τ is increased, the walk will have zero density for $\tau <\tau_{\Theta }$ and a finite density for $\tau >\tau_{\Theta }$. The density will become non-zero continuously at $\tau =\tau_{\Theta }$. The tricritical point $\tau_{\Theta }$ is identified as the collapse transition for the walk. We can calculate the correlation length exponent, ν for the model for $\tau <\tau_{\Theta }$ and $\tau =\tau_{\Theta }$. In both these cases the correlation length exponent ν may be identified with the fractal (Hausdorf) dimension of the walk through $d_{H}=1/\nu$. For $\tau >\tau_{\Theta }$ the transition seen when *K* is increased is first-order, and so the correlation length does not diverge (hence no critical exponent is defined); however, in walk models it is usual to continue to define ν via the fractal dimension. In two dimensions, the dense walk will have a dimension 2 in this phase, and so $\nu_{\mathrm{collapsed}}=1/2$. It is well known that for $\tau <\tau_{\Theta }$ the value of ν is the same as for the (non-interacting) self-avoiding walk model, $\nu_{SAW}=3/4$ [1] and at the collapse transition (Θ-point) $\nu_{\Theta }=4/7$ [7].

**Figure 2:**
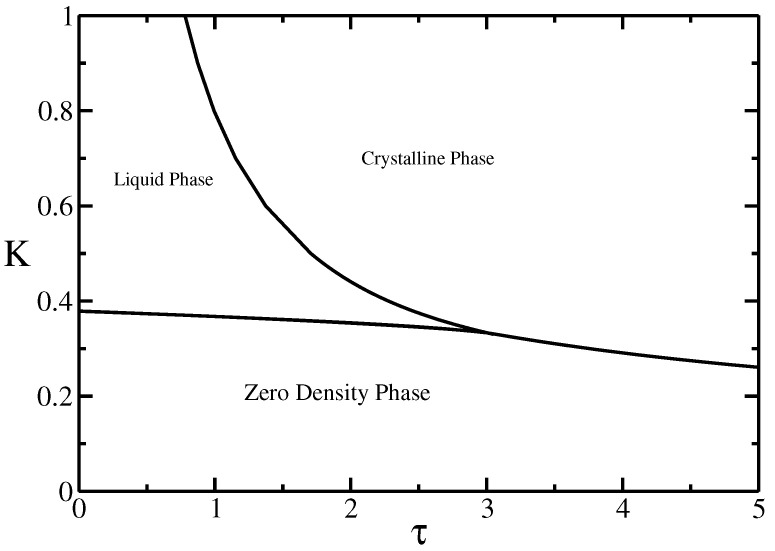
Schematic bulk phase diagram in the τ, *K* plane. *K* is the step fugacity, which controls the average length of the walk, and τ is the Boltzmann weight for the monomer-monomer interactions.

For both the ISAT model ($\tau =\tau_{c}=\tau_{x}$) and the VISAW model ($\tau =\tau_{c}$, $\tau_{x}=0$) the finite-density region is separated into two phases, separated by a transition line, ending at the collapse transition. See Figure 2. What is interesting is that both models may be naively thought to be in the same universality class as the ISAW model, but clearly are not, as the collapse point is not tricritical anymore. Foster and Pinettes [8] showed using density-matrix RG (DMRG) methods that the multi-critical point in the VISAW model is first-order in nature if the transition is approached along the $K^{⋆}\left(\tau \right)$ line, as it would be in the finite-length ensemble. This has been confirmed more recently using Monte Carlo [9]. Similar first-order behavior was observed for the ISAT [10]. Both these models are therefore different from the standard ISAW model. To understand this apparent non-universality, we need to consider that the first-order character of the collapse transition in the VSAW and ISAT models indicates that the walk is space-filling at the multi-critical (mc) collapse transition, while at the Θ-point of the ISAW model, the fractal dimension of the walk is $d_{H}$ = 7/4 < 2, and so here the walk is not space-filling, and so the walk does not see the underlying lattice for long-enough walks. The conclusion is that the Θ-point will not depend on the underlying lattice, but only the dimensionality of the problem. For the other models, however, the walks, being the same dimension as the lattice, will be sensitive to the details of the underlying lattice. Doukas et al. [11] have verified that the ISAT model on the triangular lattice has different behavior from the square lattice. In what follows we will only be considering the square lattice. The ISAT and VISAW models are models showing effects of geometric frustration, which also makes them more challenging to investigate than the traditional ISAW model. Studies of the ISAT have given rise to a plethora of apparently contradictory results [12,13,14], but it is clear that the behavior is not like the ISAW model, and a picture is beginning to emerge [10,15,16]. The VISAW model in two dimensions seems to be going in the opposite direction. While it initially seemed that the critical exponents were well understood with the Bethe-Ansatz solution [17], recent numerical evidence indicates that the situation is more complicated [18,19], although some clues about this are beginning to emerge [9].

## 2. Method

Another way of studying the phase transition in lattice models is to look at the complex zeros of the partition function. If we study the grand-canonical partition function, and look for the zeros in the complex plane, following the seminal work of Lee and Yang [20,21], it can be shown that there are no zeros on the positive real axis for finite system sizes, but that as the thermodynamic limit is approached there is a condensation of zeros which pinch the real axis at the transition point, leading to the required singular behavior in the thermodynamic limit. These zeros are known as Lee-Yang Zeros. In this article, we reexamine these models by investigating instead the finite-size scaling behavior of the zeros of the canonical (fixed-length) partition function (Fisher zeros) calculated by stochastic enumeration, using the FlatPERM method [22]. Fisher zeros are likewise expected to pinch the real axis at the correct phase transition point [23]. It is often considered that the real part of the leading zero leads to a more accurate estimate for the critical temperature and, since the imaginary part is known to vanish in the thermodynamic limit, the finite-size behavior is more easily analyzed.

The canonical partition function for walks of length *N* for all these models may be written as

(1) $$Z_{N}=\sum_{N_{I}}c_{N,N_{I}}\tau^{N_{I}},$$

where τ=exp(−βε) with ε<0 is the interaction energy and $N_{I}$ is the number of doubly visited lattice sites and $c_{N,N_{I}}$ is the number of walks of length *N* with $N_{I}$ interactions. A grand-canonical partition function may also be defined through

(2) $$\begin{array}{ccc} Z & = & \sum_{N}K^{N}Z_{N}, \end{array}$$

(3) $$\begin{array}{cc} = & \sum_{N}c_{N,N_{I}}K^{N}\tau^{N_{I}}, \end{array}$$

where *K* is the fugacity, which controls the average length of the walk through

(4) $$\langle N\rangle =\frac{\partial \ln Z}{\partial \ln K}.$$

In the limit N→∞, the walk changes from a “swollen” configuration to one which is compact, passing through the collapse temperature (or θ temperature). The swollen configuration has a Haussdorf (fractal) dimension, $d_{H}<d$, leading to a density ρ=0, for high temperatures (low-τ). The compact dimension has ρ≠0 and $d_{H}=d$. This transition may be seen to be a higher-order critical point in the grand-canonical formalism (tricritical in the ISAW model, but more complicated in the ISAT and VISAW models). We will denote the location of the multi-critical collapse transition as $\left(K^{⋆},\tau^{⋆}\right)$.

The Haussdorf dimension is defined through

(5) $$\langle R_{G}\rangle ∼N^{1/d_{H}},$$

where $R_{G}$ is the radius of gyration. By identification of the correlation function in the high-temperature expansion of the O(n) spin model in the n→0 limit, it may be seen that in the swollen phase, the correlation length exponent is $\nu =1/d_{H}$. It is usual to extend this definition to the collapse transition and low temperature phase. For $\tau >\tau^{⋆}$ the transition as *K* is increased is first-order, and so the geometric definition for ν does not give a critical exponent. It is widely accepted that at the collapse transition, the geometric definition of ν corresponds to the correct critical exponent. This appears not to be always the case, however. The collapse transition for both the ISAT and VISAW models where shown to have first-order character if approached from a specific direction [8,10], a result which has been recently confirmed by Bedini et al. [24] for the ISAT model and by Vernier et al. [9] for the VISAW model. This gives rise to a disconnection between the geometrical definition and the standard definition. Monte-Carlo simulations always calculate $\nu =1/d_{H}=1/2$ while transfer matrices have access in principle to the real correlation length exponent. For the ISAT model it is now believed that these two are the same with ν=1/2 [25], but that the VISAW is likely to have non-trivial correlation exponent value with ν=12/23≠1/2 [9].

It also explains the difference in reported exponents from Monte-Carlo methods, where ν=1/2 is calculated using Equation (5) with $\nu =1/d_{H}$, and Transfer-Matrix approaches, which deliver ν=12/23 through the finite-size scaling of the correlation length.

In this paper, we propose to reexamine this model through the Fisher zeros [23] of the canonical partition function, $Z_{N}$. These zeros, in the complex plane, are expected to accumulate and approach the real axis at $\tau^{⋆}$. For a general spin model, it is expected that the leading complex zero of the partition function will approach the real axis as follows [23]:

(6) Imτ0∼L−1ν,

(7) |Reτ0−τ⋆|∼L−1ν,

where *L* is the linear dimension.

Near the collapse transition, the singular part of the free energy is expected to scale as [26]:

(8) $$f_{s}\left(K,\tau ,L\right)=L^{-d}\tilde{f}(a_{1}|K-K^{⋆}|L^{1/\nu },a_{2}|\tau -\tau^{⋆}\left|L^{1/\nu_{2}}\right),$$

where $\nu_{2}$ is the exponent which describes the divergence of the correlation length when the tricritical point is approached for fixed $K=K_{c}$. The relevant linear dimension here is the radius of gyration ($L=R_{G}∼N^{\nu }$). When substituted into Equation (8) and using that $N∼|K-K^{⋆}{|}^{-1}$, we find:

(9) $$f_{s}\left(K,\tau ,L\right)=N^{-d\nu }\tilde{f}(b_{1},b_{2}|\tau -\tau^{⋆}\left|N^{\nu /\nu_{2}}\right).$$

the ration $\nu /\nu_{2}=\phi$ is known as the cross-over exponent, and shows that for walk models, when the zeros of the canonical partition function are considered, the real and imaginary parts of the leading zero of the partition function scales as [27]:

(10) Imτ0∼N−ϕ,

(11) |Reτ0−τ⋆|∼N−ϕ.

The same argument leads to the identification α=2−1/ϕ used in the canonical formulation of the walk models.

This method was used recently to verify $\tau^{⋆}$ and value of ϕ for the two-dimensional ISAW model using exact enumerations up to N=34 [28]. In their paper they give a value of ϕ≈0.422±0.012 in good agreement with the known exact value for the ISAW (ϕ=3/7≈0.429) but they give an estimate for $\tau_{\Theta }=2.16\pm 0.02$ which is not close to the previously calculated value $\tau_{\Theta }=1.93\pm 0.03$ [6]. This discrepancy is simply due to an error in extrapolation, as the data given in the figures are consistent with the previously known values.

## 3. Results

For the models studied in this article, the finite-size effects are more pronounced and the walk lengths available from exact enumeration calculations are not sufficient to obtain meaningful results. To overcome this difficulty, we use the FlatPERM method introduced by Prellberg and Krawczyk [22] to stochastically enumerate the coefficients of the polynomial $Z_{N}$ for sizes up to N=500 (trails) and N=1000 (VISAW). How far we went in practice depended on the model, as different models required different convergence times.

**Figure 3:**
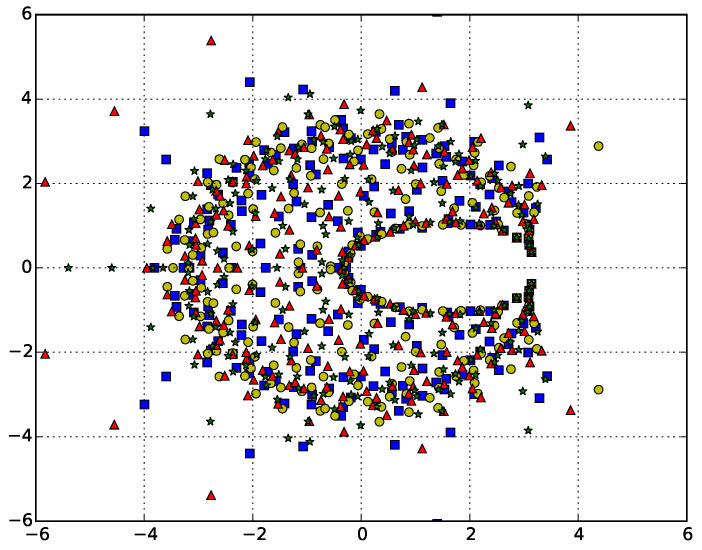
Zeros of the canonical partition function for self-avoiding trails for N=500 calculated for four different simulations. If we label the leading zero (the one closest to the positive real axis) $\tau_{0}$, the *x*-axis gives the real part of the zero and the *y*-axis the imaginary part. The complex zeros can clearly be seen to pinch the real axis near $\tau^{⋆}=3.0$, where in the thermodynamic limit the zero will sit on the axis and denote the critical point.

The canonical partition functions are calculated stochastically, resulting in stochastic variations in the estimates for their coefficients and hence in the position of the zeros. Therefore, the issue of convergence arises. Since we are interested in critical behavior, we only need to verify convergence for the zero closest to the real positive axis (the leading zero). Figure 3 shows the zeros calculated for the ISAT model with N=500 from four different simulations of $10^{8}$ iterations. While there is scatter in the zeros for the different simulations, the leading zero is superposed for the four simulations, and the second closest zero also overlaps, though with enough scatter for the different symbols to be seen. Similar results are found for the other models.

Since we are also interested in looking at the behavior of the walk in the presence of an attractive surface, we need to introduce a surface interaction energy $\varepsilon_{S}$ and associated (attractive) Boltzmann weight ω. For a given τ, as omega is increased, an infinite walk will undergo an adsorption transition at a given value of $\omega =\omega^{⋆}\left(\tau \right)$. Of particular interest is the adsorption transition which occurs simultaneously with collapse, located at $\left(\omega^{⋆},\tau^{⋆}\right)$.

To be able to investigate this, we need to extend the canonical partition function in terms of two parameters:

(12) $$Z_{N}=\sum_{N_{I},N_{S}}c_{N,N_{I},N_{S}}\tau^{N_{I}}\omega^{N_{S}},$$

with $N_{I}$ interactions, $N_{S}$ is the number of steps in the surface, and $c_{N,N_{I},NS}$ is the number of walks with *N* steps, $N_{I}$ interactions and $N_{S}$ steps in the surface.

If τ is known, then we can define the polynomial

(13) $$P_{N}\left(\omega \right)=\sum_{N_{S}}{\tilde{c}}_{N_{S}}\omega^{N_{S}},$$

where ${\tilde{c}}_{N_{S}}$ are effective coefficients, which can be calculated directly using FlatPERM in the usual way. Using the same analysis as above, the zeros of $P_{N}$ give the critical value $\omega^{⋆}$ and their finite-size behavior gives an estimate of $\phi_{S}$.

**Figure 4:**
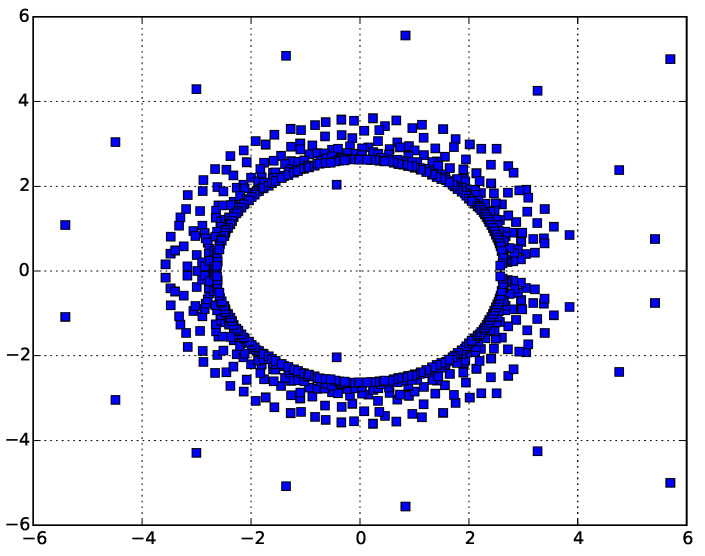
Zeros of the canonical partition function for self-avoiding trails for N=650. Again, it can clearly be seen that the leading complex zeros pinch near $\omega^{⋆}=2.45$, giving the estimate of the critical adsorption point.

For the ISAT model, the collapse transition is known exactly: $\tau^{⋆}=3$ [29]. The zeros of $P_{N}$ for the ISAT model for N=650 are shown in Figure 4.

Looking at the Figure 3 and Figure 4, we can see that the zeros pinch the real axis near the known values of $\tau^{⋆}=3$ [29] and $\omega^{⋆}\approx 2.45\pm 0.05$ [15].

**Figure 5:**
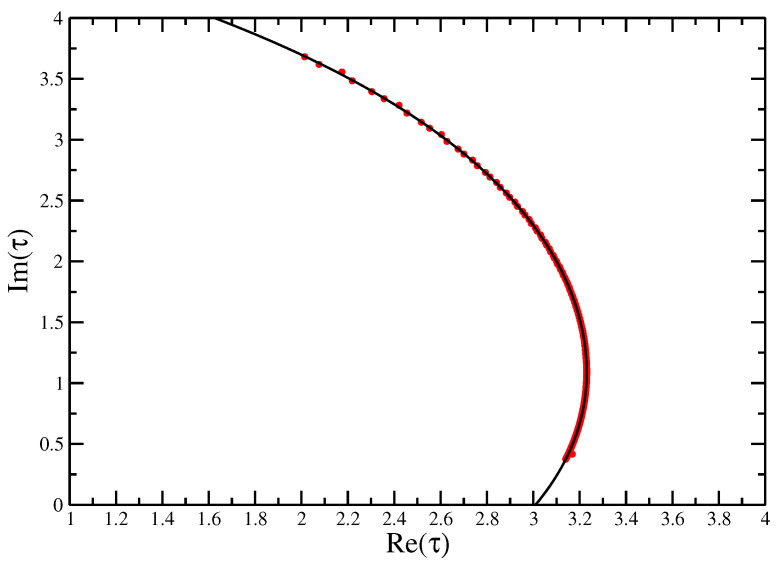
Fisher zeros for the canonical partition function of the interacting self-avoiding trail model for sizes from N=30 to N=500 fitted with a quartic function, showing the extrapolation to the critical interaction strength τ=3.01±0.01 to compare with the exact value of $\tau_{c}=3$.

Figure 5 shows the positions of the real parts of the leading zeros ($\tau_{0}$) of the partition function $Z_{N}\left(\tau \right)$. This gives finite-size estimates for $\tau^{⋆}$. It is clear that exact enumeration to a maximum length of N≈30 would be largely insufficient to estimate the value of $\tau^{⋆}$, since the evolution of $\tau_{0}$ with *N* is not monotonic. For the lengths obtained by FlatPERM we can see that the estimates extrapolate well to the previously found value, $\tau^{⋆}=3$ for the ISAT model for large enough *N*. The points are fitted following Taylor and Luettmer-Strathmann [30], who fitted the real part of the zero as a polynomial of the imaginary part. As the imaginary part goes to zero as the length of the walk goes to zero, the asymptotic estimate is given by the constant part of the constructed polynomial. In this case, giving $\tau_{\mathrm{est}}^{⋆}=3.01\pm 0.01$, which compares well to the exactly known value, $\tau^{⋆}=3$. In the models presented here we found the best fit occurred when we fitted the real part of the leading zero to a quartic polynomial in the imaginary.

While the real part of the leading zero behaves in a non-monotonic fashion, making it difficult to reach an asymptotic regime from which to calculate ϕ, the imaginary part is better behaved. Additionally, the imaginary part extrapolates to zero in the limit N→∞. We can try and estimate ϕ in two different ways. The first is to take Equation (10) which gives:

(14) $$Im\left[\tau_{0}\left(N\right)\right]\approx AN^{-\phi }+\mathrm{higher}\;\mathrm{order}\;\mathrm{terms}.$$

Eliminating *A*, and ignoring the higher-order terms leads to an effective value $\phi_{\mathrm{eff}}$

(15) $$\phi_{\mathrm{eff}}=\frac{Im\left[\tau_{0}\left(N_{1}\right)\right]/Im\left[\tau_{0}\left(N_{2}\right)\right]}{N_{1}/N_{2}}.$$

**Figure 6:**
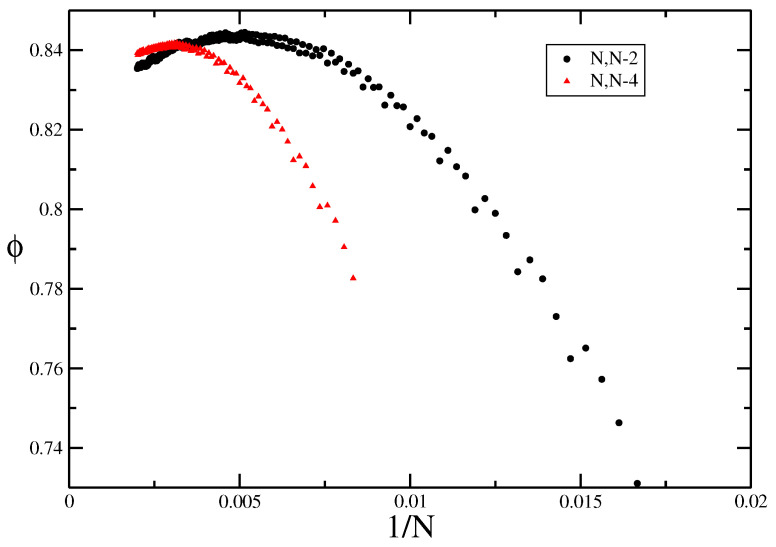
The effective bulk cross-over exponent for the trails model, $\phi_{\mathrm{eff}}$ plotted against 1/N where the exponent is calculated using two lattice sizes. Due to parity effects, we have looked at two cases: $N1=N,N_{2}=N-2$ and $N_{1}=N,N_{2}=N-4$.

In the limit $N_{1},N_{2}\to \infty$, we would expect $\phi_{\mathrm{eff}}\to \phi$. Since there are strong parity effects in these models, it is necessary to take $N_{1}$ and $N_{2}$ to be both even or both odd. In Figure 6 we show two series of estimates, one with $N_{1}=N,N_{2}=N-2$ and the other $N_{1}=N,N_{2}=N-4$. Both sets of curves plotted against 1/N lead to estimates of ϕ=0.83±0.01. The quality of this method depends crucially on the coefficients being calculated very accurately, and so is sensitive to the errors induced by stochastic enumeration. This method did not work so well for the other models.

**Figure 7:**
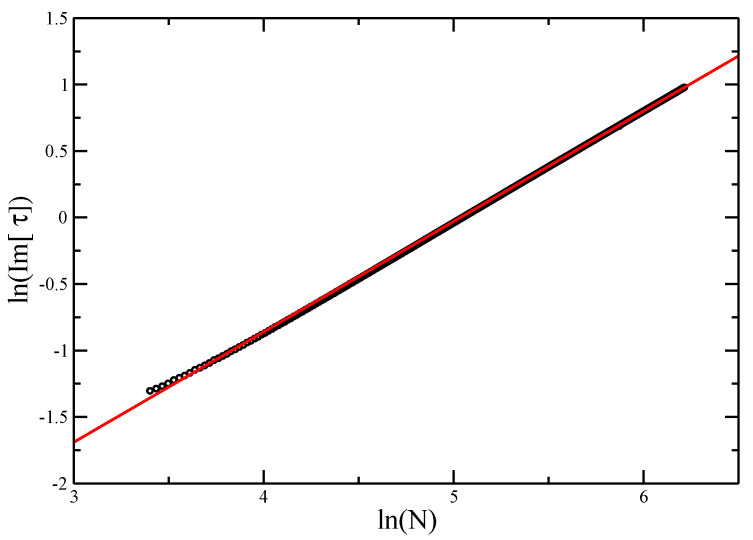
logIm(τc) vs. logN for Trails O(n). The slope of the best fit gives an estimate of ϕ=0.83±0.02.

Otherwise, we can estimate directly by taking the log of Equation (14), giving $\log(Im\left[\tau_{0}\left(N\right)\right])=-\phi \log\left(N\right)+A+$ (h.o. terms). For the ISAT model, this is shown in Figure 7, and leads to an estimate ϕ≈0.83 as well. As the line is not quite straight, the quality of the fit depends on only using the most asymptotic part of the line. There is a risk that if the length of walk considered is not sufficient, the estimate might still be evolving. This appears not to be the case here.

**Figure 8:**
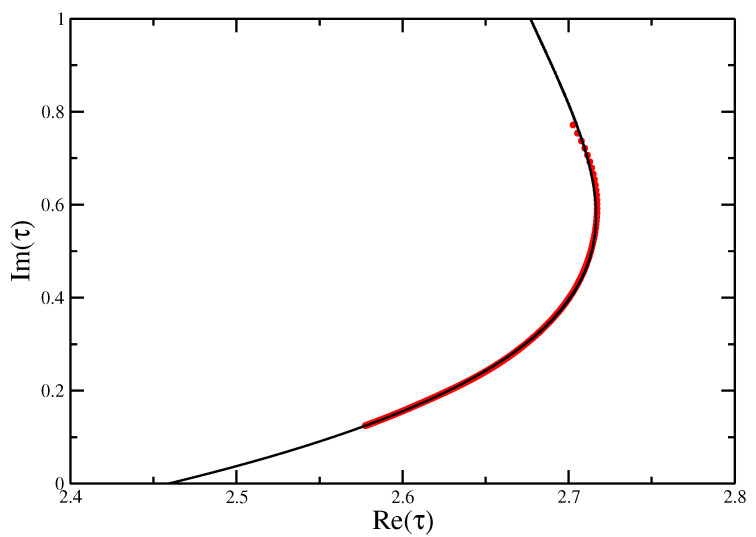
A cubic fit of the Re(ωc) against Im(ωc) for the surface interaction of Trails at the special surface transition with $\tau =\tau^{⋆}=3$.

Figure 8 shows the finite-size estimates of the zeros for $P_{N}$ go the ISAT model with $\tau =\tau^{⋆}$, giving an estimate of $\omega^{⋆}\approx 2.45\pm 0.02$, to be compared with 2.45±0.05 found from transfer matrices [15]. Fitting the log-log curve with a straight line gave an estimate of $\phi_{S}\approx 0.52\pm 0.01$. In reference [15], it was found that $\nu_{S}=1.275\pm 0.005$, which along with the geometric exponent ν=1/2 would give $\phi_{S}=0.393\pm 0.002$. These results differ significantly, but $\phi_{S}$ is one of the more difficult exponents to estimate. The location of the critical point is however in good agreement.

**Figure 9:**
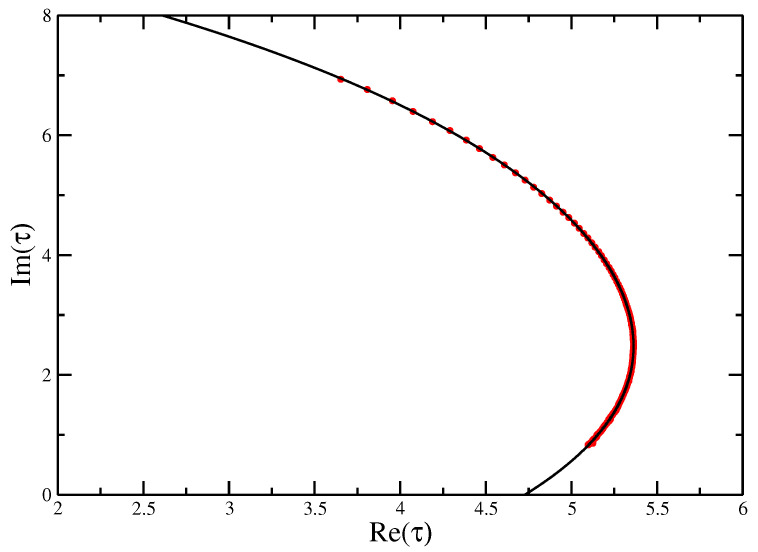
Fisher zeros for the canonical partition function of the VISAW model fitted with a quartic function, showing the extrapolation to the critical interaction strength τ=4.76±0.04 to compare with the value of $\tau_{c}=4.69\pm 0.01$ calculate from Transfer Matrices and DMRG.

**Figure 10:**
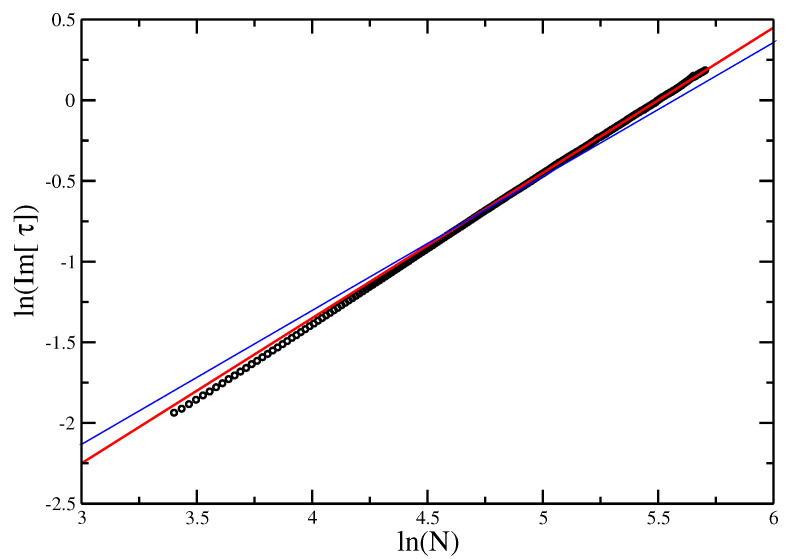
logIm(τc) vs. logN for the VISAW model. The red line gives the slope of best fit, giving an estimate of ϕ=0.89±0.01. The points appear to lie on a straight line, and the blue line (of slope 0.83±0.02, as found for the Trails) is shown as reference. While it is possible that the line evolves further to reach the slope of the blue line, we would argue that this supports the hypothesis that the ISAT and the VISAW are in different universality classes.

Figure 9 and Figure 10 give the locus of the leading zeros and the imaginary part of $\tau_{0}$ vs. *N* on a log-log plot for the VISAW model. These give estimates $\tau^{⋆}=4.76\pm 0.04$ and ϕ=0.89±0.01 (compared with 4.69±0.01 found using DMRG [8] the result here seems a little high).

Comparing the estimates ϕ=0.89±0.01 for VISAW and ϕ=0.83±0.01 for the ISAT model, we find that these two model have different cross-over behavior, unlike that found by Bedini et al. [24], who found the two values to be very close at ϕ≈0.84 ($\phi_{SAT}=0.84\pm 0.3$). The error bars are estimated using reasonable extremal fits. There is always a possibility that the slope of the line is still evolving, so, in Figure 10, we show lines of the two slopes and argue it is unlikely that asymptotically the line will move sufficiently to reach the required slope for $\phi_{VISAW}=\phi_{ISAT}$, although this cannot be excluded.

The interesting feature of the VISAW generalized to include stiffness (p≠1) it that it contains an integrable point [5]. It was expected that the critical behavior of this integrable point was the same as the general VISAW at the collapse transition, and in particular the VISAW model with p=1. The advantage numerically of investigating this integrable point is that its location is known exactly: $\tau^{⋆}=2.630986\cdots$, $p^{⋆}=0.275899\cdots$ and $K^{⋆}=0.446933\cdots$. The Bethe-Ansatz led to a conjecture on the exact exponents at the critical point, notably the correlation length exponent ν=12/23 and the cross-over exponent ϕ=10/23 [17]. These exponents were conjectured true for the standard VISAW model as well, but have never been observed numerically, either by transfer matrix/DMRG [5,8] or in Monte Carlo [24]. Indeed, the behavior at the integrable point seems to be very different from the normal VISAW model. Numerically, both DMRG [18] with a surface and Monte Carlo [19] investigations at the integrable point find strong evidence of ν≈0.576 being close or the same as the standard ISAW model. This has been investigated by Vernier, Jacobsen and Saleur [9], who have identified the integrable point with an infinitely critical point, and have argued that this leads to finite-size correction terms which would make the identification of the exact exponents extremely difficult numerically. While it is not yet fully understood how this point fits in with the rest of the phase diagram, it is unclear if the standard VISAW is in the same universality class.

**Figure 11:**
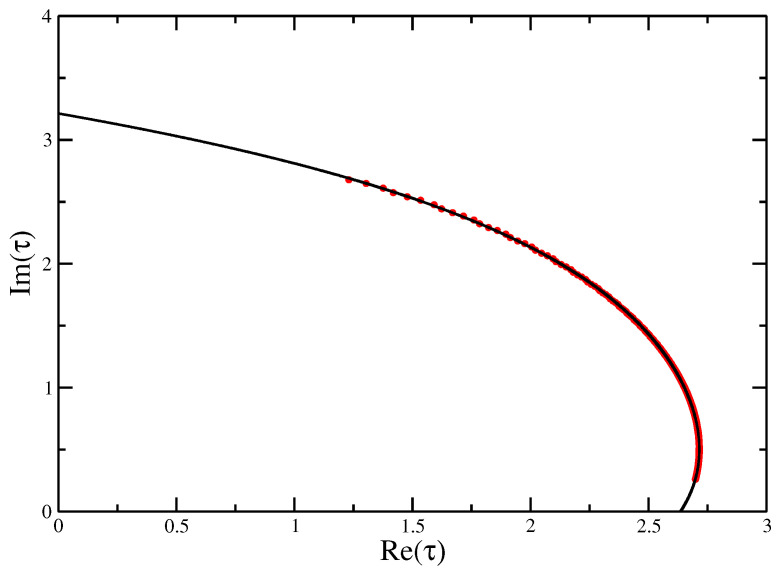
Fisher zeros for the canonical partition function of the Integrable VISAW model at the integrable point fitted with a quartic function, showing the extrapolation to the critical interaction strength τ=2.64±0.02 to compare with the exact value of $\tau_{c}=2.630986\cdots$.

**Figure 12:**
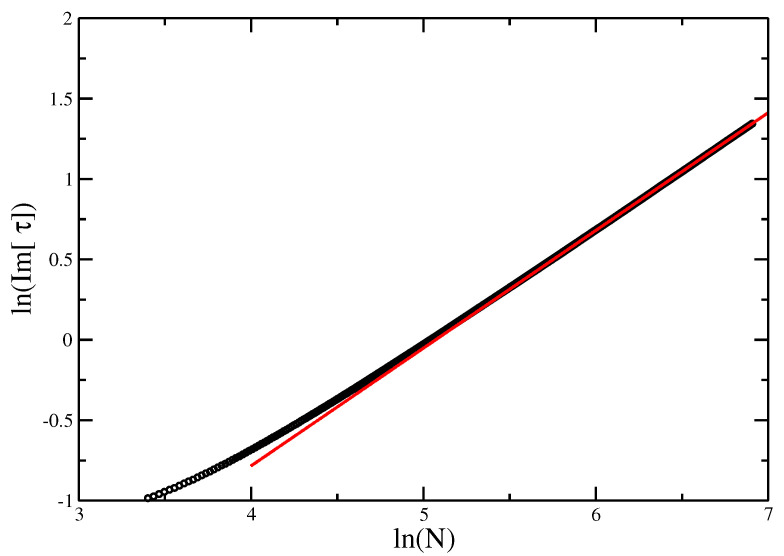
logIm(τc) vs. logN for the Integrable VISAW model. The slope of the best fit gives an estimate of ϕ=0.73±0.01.

**Figure 13:**
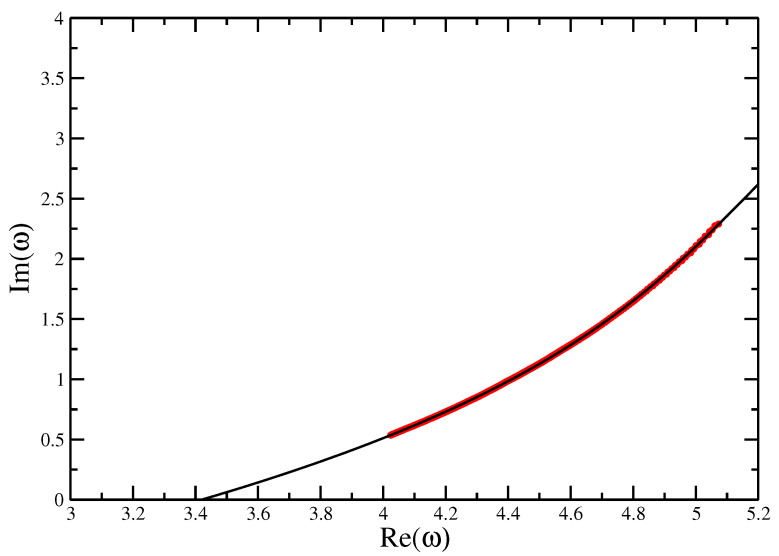
A cubic fit of the Re(ω⋆) against Im(ω⋆) for the surface interaction of the Integrable VISAW model at the collapse transition with $p=p^{⋆}$ and $\tau =\tau^{⋆}$.

The zeros of the partition function again reproduce the location of the collapse transition for this integrable point very well (see Figure 11) as $\tau^{⋆}\approx 2.64\pm 0.02$ (cf. exact value of 2.630986...), and the best fit for the cross-over exponent, shown in Figure 12, is ϕ=0.73±0.01, although, in light of the preceding discussion, this value needs to be taken with a lot of caution about what it actually means. Putting in the $\tau^{⋆}$ and $p^{⋆}$ for the integrable point, it was possible to identify $\omega^{⋆}=3.42\pm 0.04$ at the adsorption transition to be compared with a value of $\omega^{⋆}=3.41\pm 0.01$ from transfer matrices [18]. A cubic fit of the real part of the finite size estimates for $\omega^{⋆}$ as a function of the imaginary part is shown in Figure 13, showing the extrapolation to $\omega^{⋆}\approx 3.42$.

## 4. Conclusions

In this article, we looked at using the stochastic enumeration of series to generate partition functions of finite-length walks to investigate performance in locating the critical points (surface and bulk) and to calculate cross-over exponents. The cross-over exponents compared favorably with values found by other methods, notably looking at the scaling behavior of the specific heat. The models presented here are numerically particularly difficult, and the complex zeros performed very well in identifying accurately the transition points for the different models. This method has an advantage that for a given size, the location of the leading zero is well defined and less sensitive to error than, say, the peak of a specific heat curve. It provides direct access to the finite-size scaling behavior. We believe it provides a useful tool to exploit the FlatPERM method in investigating lattice walk models.

With good quality values of the zeros (Yang-Lee and Fisher) for different sizes of polymer length should enable access to more information about the models being studied, see for example E J Janse van Rensburg [31]. This, along with a detailed finite-size study of the adsorption transitions, will be the object of further work. Similar questions on the accuracy of the surface exponents exist for the three-dimensional self-avoiding walk model, see for example reference [32] and references therein.
